# Comparative Analysis of Muscle Atrophy During Spaceflight, Nutritional Deficiency and Disuse in the Nematode *Caenorhabditis elegans*

**DOI:** 10.3390/ijms241612640

**Published:** 2023-08-10

**Authors:** Ban-seok Kim, Alfredo V. Alcantara, Je-Hyun Moon, Atsushi Higashitani, Nahoko Higashitani, Timothy Etheridge, Nathaniel J. Szewczyk, Colleen S. Deane, Christopher J. Gaffney, Akira Higashibata, Toko Hashizume, Kyoung-hye Yoon, Jin I. Lee

**Affiliations:** 1 Division of Biological Science and Technology, College of Science and Technology, Yonsei University, Wonju 26493, Republic of Korea; corona_a@hanmail.net (B.-s.K.); alfred.alcantarajr@gmail.com (A.V.A.J.); dmjh813@naver.com (J.-H.M.); 2 Graduate School of Life Sciences, Tohoku University, Sendai 980-8577, Japannaho@ige.tohoku.ac.jp (N.H.); 3 Department of Sport and Health Sciences, College of Life and Environmental Sciences, University of Exeter, Exeter EX1 2LU, UK; t.etheridge@exeter.ac.uk (T.E.); c.s.deane@soton.ac.uk (C.S.D.); 4 Ohio Musculoskeletal and Neurological Institute, Heritage College of Osteopathic Medicine, Ohio University, Athens, OH 45701, USA; szewczyk@ohio.edu; 5 Human Development & Health Faculty of Medicine, University of Southampton, Southampton General Hospital, Southampton SO16 6YD, UK; 6 Lancaster Medical School, Health Innovation One, Sir John Fisher Drive, Lancaster University, Lancaster LA1 4AT, UK; c.gaffney@lancaster.ac.uk; 7 Human Spaceflight Technology Directorate, Japan Aerospace Exploration Agency, Tsukuba 305-0047, Japan; 8 Advanced Engineering Services Co., Ltd., Tsukuba 305-0032, Japan; 9 Department of Physiology, Mitohormesis Research Center, Yonsei University Wonju College of Medicine, Wonju 26426, Republic of Korea; kyounghyeyoon@yonsei.ac.kr

**Keywords:** *C. elegans*, space, microgravity, muscle atrophy, calpain

## Abstract

While spaceflight is becoming more common than before, the hazards spaceflight and space microgravity pose to the human body remain relatively unexplored. Astronauts experience muscle atrophy after spaceflight, but the exact reasons for this and solutions are unknown. Here, we take advantage of the nematode *C. elegans* to understand the effects of space microgravity on worm body wall muscle. We found that space microgravity induces muscle atrophy in *C. elegans* from two independent spaceflight missions. As a comparison to spaceflight-induced muscle atrophy, we assessed the effects of acute nutritional deprivation and muscle disuse on *C. elegans* muscle cells. We found that these two factors also induce muscle atrophy in the nematode. Finally, we identified *clp-4*, which encodes a calpain protease that promotes muscle atrophy. Mutants of *clp-4* suppress starvation-induced muscle atrophy. Such comparative analyses of different factors causing muscle atrophy in *C. elegans* could provide a way to identify novel genetic factors regulating space microgravity-induced muscle atrophy.

## 1. Introduction

As more countries and companies advance spaceflight technology, space travel, habitation and tourism are becoming more common. Whereas space travel was reserved for astronauts in the past, today, private citizens can experience the space environment. However, with the increasing number of spaceflights and deeper ventures into space, understanding the changes and dangers that the space environment and microgravity pose to the human body has become more pertinent. Several studies have demonstrated that microgravity induces muscle atrophy in space [1,2], which is a serious concern for future long-term space habitation. To address this issue, various approaches have been proposed, such as implementing effective exercises in space [3]. However, comprehensive research is still urgently needed to uncover the fundamental causes and mechanisms behind microgravity-induced muscle atrophy.

Muscle atrophy can occur due to various factors other than space microgravity, including disease, disuse, malnutrition and aging [4,5,6,7,8]. Studies in these areas have revealed various genetic factors that are involved in muscle atrophy, such as MuRF1, atrogin-1, dystrophin and calpains [9,10,11]. However, our knowledge of the underlying mechanisms of muscle atrophy remains incomplete and the connection to space microgravity-induced muscle atrophy is not known.

The bacterivorous nematode *Caenorhabditis elegans* has been a premier genetic model animal for over five decades due to its fast rate of growth, ease of use and other characteristics [12,13]. *C. elegans*’ body wall muscle, similar to vertebrate skeletal muscle, propels the movement of the worm, and the development, structure and maintenance of *C. elegans* muscle has been thoroughly studied for decades [14]. *C. elegans* experiments have also been conducted in space, where worms are most effectively cultured in a liquid medium [15,16,17,18]. Analyses of RNA and protein expression in space-flown worms from these missions revealed a significant decrease in the expression levels of several muscle-related genes, including *myo-3* and *unc-54* (myosin heavy chain orthologs), as well as transcription factors like *hlh-1* (MyoD ortholog) [19,20]. In addition, the movement of worms in space was slower than on earth [20].

These studies provide evidence that *C. elegans* muscle, like human muscle, may undergo atrophy during spaceflight. In combination with powerful *C. elegans* genetic techniques, spaceflight studies with *C. elegans* could identify genetic and molecular targets of microgravity-induced muscle atrophy and create a platform to develop novel therapies against muscle atrophy for astronauts and space travelers. In this study, we sent *C. elegans* aboard the International Space Station (ISS) and found that body wall muscle becomes smaller in the space environment. Given that further space mission experiments are rare and limited, we decided to use a comparative approach with other muscle atrophy factors to gain a better understanding. In our experiments, we specifically focused on disuse and nutritional deprivation as conditions for comparison and found that both factors cause muscle atrophy, as in spaceflight. Finally, we identified a gene, *clp-4*, that encodes a calpain protease that promotes muscle atrophy.

## 2. Results

### 2.1. Muscle Atrophy in Space-Microgravity-Exposed C. elegans

**Figure 1:**
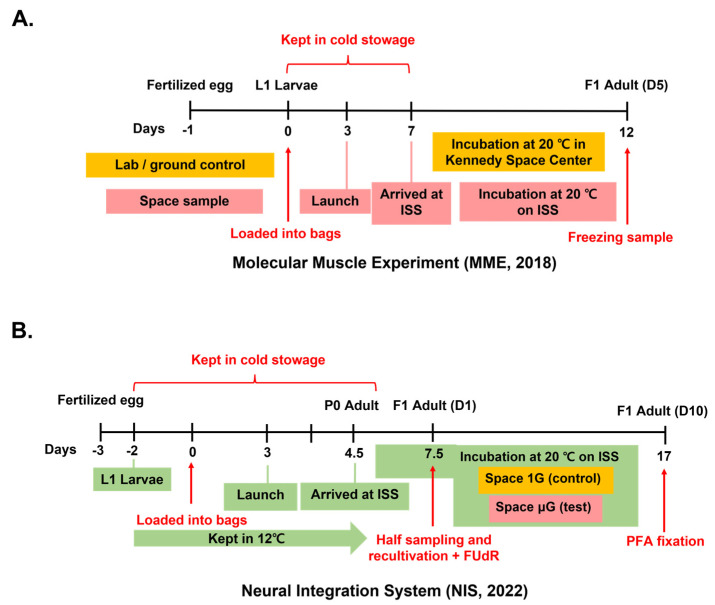
Space experiment protocols of two space missions. Red indicates common events in both control and space samples. The events only occurring for control samples are indicated in the orange box, and space (test) samples are indicated in the pink box. The eggs were collected by bleaching worms. (**A**) MME space mission timeline. Modified from Laranjeiro et al., 2021. (**B**) NIS space mission timeline. The green box indicates the events occurring for both space 1G (control) and space μG (test).

**Figure 2:**
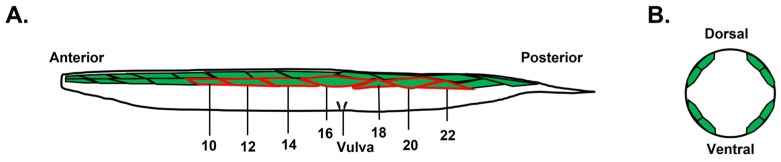
Illustration of body wall muscles in *C. elegans*. (**A**) Dorsal lateral body wall muscles are arranged as staggered pairs. Seven dorsal lateral body wall muscle cells numbered 10, 12, 14, 16, 18, 20 and 22 are highlighted with a red border. (**B**) Cross-sectional illustration of the body of *C. elegans*. Four longitudinal bundles which consist of 23 or 24 paired muscles are located in four quadrants.

To better understand how space microgravity affects *C. elegans* muscle, we collaborated with the European Space Agency (ESA) to send *C. elegans* to the ISS. Young adult worms from the parental generation arrived on the ISS and remained in microgravity conditions for approximately 5 days, laying eggs to generate an F1 generation that was born aboard the ISS (Figure 1A). Thus, the majority of the worms from these samples spent their entire life in space microgravity conditions. We analyzed a standard wild-type (WT) *C. elegans* strain N2 [12], and the *unc-70 (e524)* mutant strain, which is a weak mutant of the β-spectrin gene. *unc-70 (e524)* mutants were reported to have a smaller longitudinal muscle area (Figure 2) than the wild type (WT) [21], but the overall structure of the muscle is normal, unlike that of stronger β-spectrin null mutants [22,23]. In addition, ground controls were maintained in similar conditions to those maintained for the space samples at NASA Kennedy Space Center.

**Figure 3:**
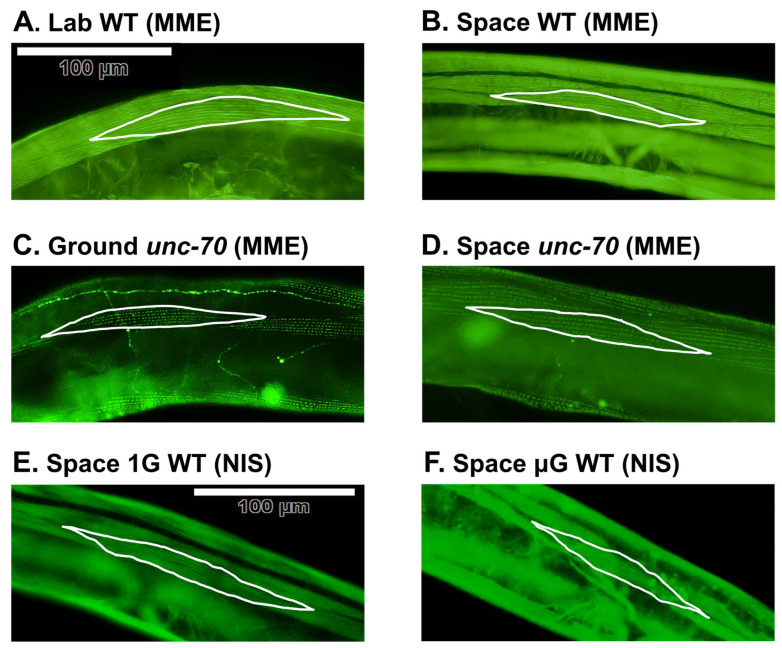
Stained muscles of MME and NIS samples. (**A**–**D**) MME mission samples stained with Alexa Fluor™ 488 phalloidin. WT (MME) is N2; *juIs76 (unc-25p::GFP)*, that is, a wild-type N2 strain expressing GFP transgenically in the DD/VD motor neurons. *unc-70* is *unc-70*; *juIs76 (unc-25p::GFP)*, that is, an *unc-70 (e524)* mutant strain expressing GFP transgenically in the DD/VD motor neurons. DD/VD neurons expressing GFP can be seen in (**C**,**D**). (**E**,**F**) NIS mission samples stained with phalloidin. WT (NIS) is the N2 strain. Body wall muscle #16 is highlighted with a white border in all pictures. The bar in (**A**) (=100 μm) is applied to (**A**–**D**), and the bar in (**E**) (=100 μm) is applied to (**E**,**F**).

After staining samples with phalloidin, we observed that the actin structure and sarcomere patterns of the muscle cells in the WT and *unc-70* were indistinguishable in the space and control samples (Figure 3A–D). Since the wild-type ground control samples we received were not in an adequate state for analysis and quantification, we instead generated laboratory control samples (lab WT) using the same flight conditions as the MME mission (Figure 1A and Figure 3A).

We noticed clear differences in the size of the muscle cells in space compared to the controls, so we measured the area of muscle cells in each of the worms. *C. elegans* body wall muscle comprises 95 rhomboid-shaped muscle cells arranged in four longitudinal bundles, two dorsally and two ventrally, spanning most of the anterior–posterior axis of the worm ([24]; Figure 2). We did not observe any specific differences between muscle cells in the dorsal and ventral bundles in the space and control samples, so we focused our analysis on the dorsal lateral body wall muscle cells. The 24 muscle cells in a single bundle greatly vary in size across the longitudinal axis [21]. Thus, we set out to analyze a total of seven body wall muscle cells per animal: cells 10, 12, 14, 16, 18, 20 and 22 (Figure 2A). An accurate measurement of muscle cell size would require measurement of the longitudinal area as well as cell height. However, since the height of the cells is much smaller than the longitudinal area [25] and accurate measurements of cell height would require electron microscopy analysis, we approximated muscle cell size by measuring only the longitudinal area of the cell, as previously shown [21].

**Figure 4:**
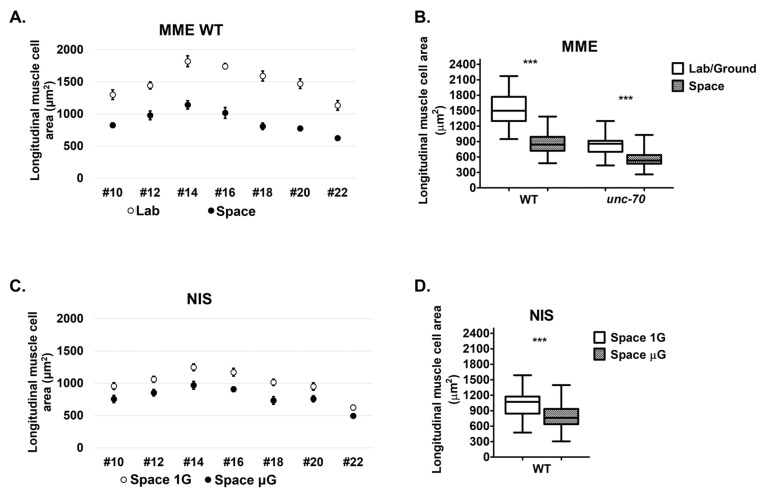
Longitudinal muscle cell area of samples from two space missions. (**A**,**C**) Longitudinal muscle cell area of #10, #12, #14, #16, #18, #20 and #22 muscle cells. (**B**,**D**) Net longitudinal muscle cell area of 7 dorsal lateral body wall muscles. Error bars indicate standard error. Statistical significance was determined via Student’s *t*-test. *** indicates statistical significance *p* < 0.001.

We found that the average dorsal muscle longitudinal cell area ranged from 1135 μm^2^ in cell 22 to 1821 μm^2^ in cell 14 with an average size of 1501 μm^2^ in the WT (Figure 4A,B). Interestingly, the muscle cell sizes in the space samples were clearly smaller, ranging from 626 μm^2^ in cell 22 to 1142 μm^2^ in cell 14 with an average size of 891 μm^2^ in the WT (Figure 4A,B). The control *unc-70* mutant muscle cells were smaller than the WT, as expected [21]. As in the WT, we also observed smaller-than-normal muscle cells in the space samples, with an average decrease of 37.3% in *unc-70* mutant muscle cell area in space. The atrophy in longitudinal muscle area was observed consistently in all seven dorsal lateral body wall muscle cells (Figure 4A). Thus, the space environment causes atrophy of body wall muscle cells independently of *unc-70* β-spectrin mutation.

We had an opportunity to confirm our results from the MME space mission during the Neural Integration System (NIS) mission. In contrast to the MME experiments, the NIS experiments included a 1 G centrifuge aboard the ISS, which generated a space 1 G control in addition to the space 0 G experimental samples. Similar to the MME experiments, we observed a consistent decrease in longitudinal muscle cell areas for all seven dorsal lateral body wall muscle cells, and an overall decrease in the average muscle cell area of 23.4% in space 0 G compared to space 1G (Figure 4C,D). Taken together, exposure to space microgravity correlates with a decrease in body wall muscle cell size.

**Figure 5:**
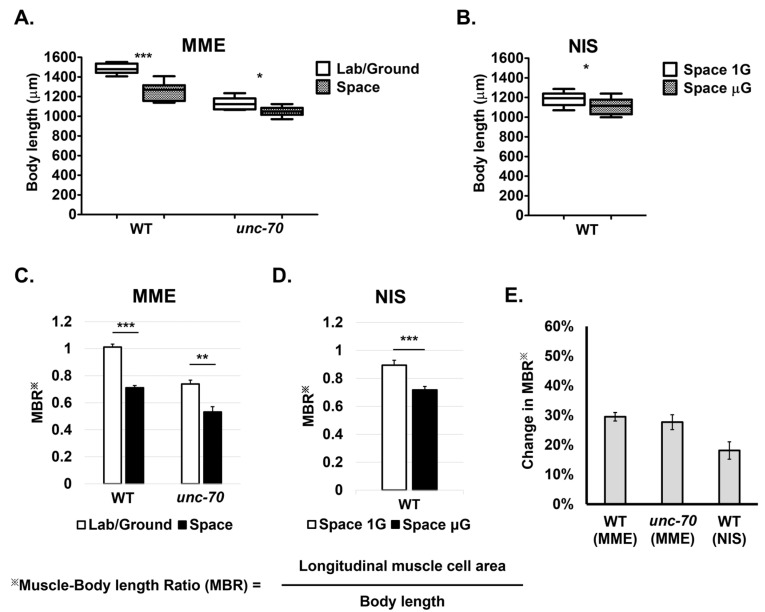
Body length and normalized muscle-to-body length ratio (MBR) for two space mission samples. (**A**,**B**) Body length for MME samples (**A**) and NIS samples (**B**). (**C**,**D**) MBR comparison to normalize muscle cell area with body length. ※ indicates MBR calculation which is indicated below the graphs. (**E**) Percent change in MBR between control and test samples to determine extent of muscle atrophy. Error bars indicate standard error. Statistical significance was determined via Student’s *t*-test. * and ** and *** indicate statistical significance at *p* < 0.05, *p* < 0.005 and *p* < 0.001, respectively.

Previous studies have shown that *C. elegans* overall body length is smaller in space [20,26]. Thus, one possibility is that the smaller muscle area observed in space may be due to growth limitations in the space environment and overall smaller body size. To ascertain this possibility, we determined the body length in the control and space samples, and then normalized our muscle cell area data with body length by calculating a muscle-to-body length ratio (MBR). We confirmed the previous reports that body length is significantly smaller in space in both the MME and NIS experiments and for both the WT and the *unc-70* mutant strain (Figure 5A,B). Next, we calculated the muscle-to-body length ratio for the MME and NIS experiments (Figure 5C,D) and found a decrease in the MBR of 29.7% and 27.4% for the WT and *unc-70*, respectively, in the MME experiment, and a 19.8% decrease in the WT in the NIS experiment (Figure 5E). This suggests that the level of muscle shrinkage was much higher than the decrease in body length and indicates the occurrence of microgravity-associated muscle atrophy.

### 2.2. Muscle Atrophy in Nutritional Deprivation and Disuse Conditions in C. elegans

Genetic analysis using *C. elegans* affords us an opportunity to understand the cellular and molecular mechanism of how space microgravity induces muscle atrophy in the body wall muscle cells. However, sampling and other restrictions in space experiments, together with the few space mission opportunities, are limiting factors in conducting further genetic analysis in space. Muscle atrophy is not limited to space microgravity, and other factors can induce smaller muscle, including nutritional deprivation and muscle disuse [4,5,6]. Thus, we applied these two factors to determine whether they also induce muscle atrophy in *C. elegans*.

**Figure 6:**
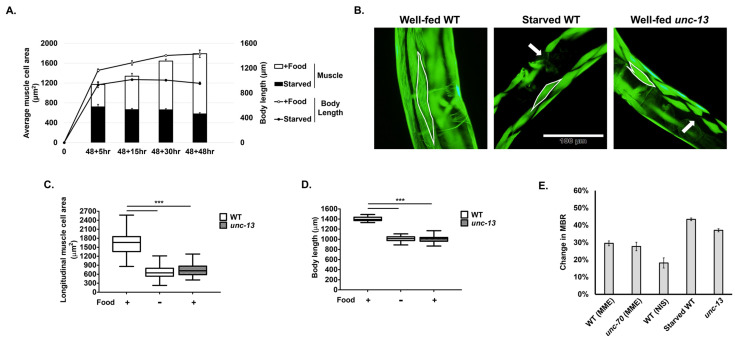
Effects of nutritional deprivation and disuse on longitudinal muscle cell area, body length and MBR. (**A**) Changes in average muscle cell area and body length over time during starvation. X-axis indicates 48 h of feeding from L1 larvae and then additional time in the presence or absence of LabTie freeze-dried OP50 as a food. (**B**) Phalloidin-stained muscle images of well-fed WT, starved WT and well-fed *unc-13* mutant. Body wall muscle #16 is highlighted with white border in all pictures. Occasionally, some muscles lose their rhomboid shape (arrow). Bar in (**B**) =100 μm. (**C**,**D**) Measurement of muscle cell area and body length. Food (+) indicates that food was provided for the experiment, whereas (−) indicates that food was not provided. (**E**) Percent change in the MBR in different muscle atrophy conditions (space, starvation, disuse). To compare values, the results of space missions shown in Figure 5E are represented together. Error bars indicate standard error. Statistical significance was determined via one-way ANOVA and Tukey’s post hoc analysis. *** indicates statistical significance *p* < 0.001.

To determine whether nutritional deprivation is associated with muscle atrophy, we provided *E. coli* food to developmentally synchronized L1-stage larvae for 48 h, and then removed the food, starving the worms for 5 to 48 h. Normally, in the presence of ample nutrition, *C. elegans* continues to grow in both body length and longitudinal muscle area from 53 h to 96 h (Figure 6A). However, upon starvation, body length growth slowed substantially, and muscle cell size stopped increasing (Figure 6A) and appeared to shrink (Figure 6B), eventually leading to the death of some worms after 48 h. After 30 h of starvation, muscle cell area and body length were greatly reduced in experimental worms compared to those undergoing continual feeding (Figure 6C,D). More importantly, we found a large reduction in the MBR of over 40% by 30 h in starved animals (Figure 6E). Hence, nutritional deprivation correlates with muscle atrophy in *C. elegans*, as in most other animals [5,6,27].

Muscle disuse, whether due to disease, denervation experiments or hindlimb suspension experiments, is known to cause muscle atrophy [4,9,28,29]. In *C. elegans*, we used *unc-13* strain mutants to replicate muscle disuse. The *unc-13* gene encodes the *C. elegans* orthologue of the MUNC-13 mammalian protein, which serves a function in pre-synaptic neurotransmitter synaptic vesicle release [30,31]. The severe *unc-13 (s69)* mutant does not display any evoked synaptic activity, including any neuromuscular synaptic activity, and is almost completely paralyzed but overall can eat, develop and survive rather normally [12,30,32], unlike other mutants of key genes involved in the *C. elegans* neuromuscular junction. We found that the body wall muscle of *unc-13* mutants was smaller than the muscle of well-fed WT strain worms, displaying an average area of 665 μm^2^, which is comparable in size to the area of muscle in starved animals at 741 μm^2^ (Figure 6B,C). The body length of *unc-13* mutants was also smaller than that of well-fed WT *C. elegans* (Figure 6D), but the MBR decreased nearly 40% in *unc-13* mutants compared to WT animals, indicating substantial muscle atrophy due to disuse comparable to atrophy during nutritional deprivation (Figure 6E).

### 2.3. clp-4 Promotes Muscle Atrophy during Nutritional Deprivation

**Figure 7:**
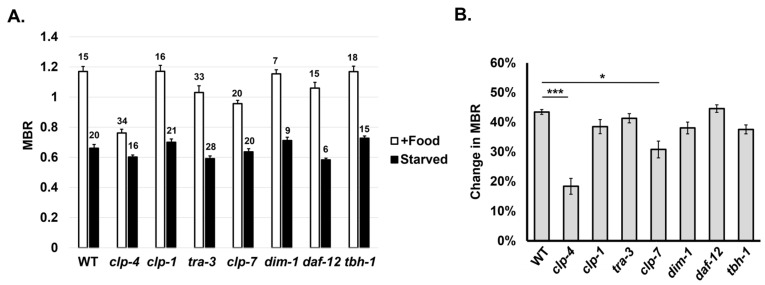
Candidate genetic screen for factors that can alter starvation-induced muscle atrophy. (**A**) MBR of WT and candidate mutants in fed and starved conditions. Numbers on the bars indicate the number of worms. (**B**) Percent change in MBR in fed vs. starved conditions for each strain. The bars that have the same lower-case alphabets can be grouped statistically. Error bars indicate standard error and statistical analysis as determined with one-way ANOVA and Tukey’s post hoc analysis. * and *** indicate statistical significance *p* < 0.05 and *p* < 0.001, respectively.

To identify genetic factors that induce muscle atrophy, we conducted a candidate genetic screening using *C. elegans* mutants. Among the three experimental conditions of muscle atrophy that we have identified, starvation was the most straightforward experimentally. Therefore, we based our genetic screening on the nutritional deprivation method and evaluated the muscle atrophy of various mutant lines by determining the change in the MBR after 30 h of starvation. We first tested mutants of *dim-1*, which encodes a muscle-expressed immunoglobin protein. DIM-1 is known to be involved in the maintenance of muscle structure, and loss of *dim-1* protects against disruption of attachment complexes and associated phenotypes [33,34]. In addition, *dim-1* expression was decreased during spaceflight in two independent space experiments [20]. However, in *dim-1* starved animals, we observed smaller muscle and a decreased MBR similar to that of WT animals, indicating that the loss of *dim-1* does not alter muscle atrophy during nutritional deficiency (Figure 7A,B). We next tested genes encoding members of the calpain family of calcium-dependent cysteine proteases. Various calpain proteins have differential functions in muscle sarcomere and muscle mitochondrial development as well as attachment complex formation [34]. We found that mutation of the calpain genes *clp-1* and *tra-3* did not alter starvation-induced muscle atrophy, and *clp-7* mutation had a mild effect (Figure 7A,B). However, disruption of *clp-4* had a profound effect, decreasing the MBR of WT animals from 43.6% to only 21% in *clp-4* mutants (Figure 7A,B). Finally, we also observed muscle atrophy in *daf-12*- and *tbh-1-*deficient animals. DAF-12 and TBH-1 are known to protect *C. elegans* from starvation-induced death [35]. However, both *daf-12* and *tbh-1* mutants showed a similar decrease in the MBR during starvation, indicating that starvation-induced muscle atrophy may be independent of starvation tolerance pathways (Figure 7A,B). Overall, we found that the calpain gene *clp-4* promotes muscle atrophy, and inhibition of *clp-4* can protect *C. elegans* muscle from complete starvation-induced muscle atrophy.

## 3. Discussion

Long-term space travel, tourism and habitation are on the horizon for humankind, but microgravity poses significant health and biological problems that must be overcome. Space-induced muscle atrophy has been demonstrated in a range of organisms including humans, rodents and primates, and now includes the nematode *C. elegans* [36]. However, identifying the molecular and genetic underpinnings of microgravity-induced muscle atrophy and developing therapies to prevent it are hampered by the difficulty of conducting practical and effective experiments in space. Using *C. elegans* as a model animal for muscle atrophy affords us the opportunity to conduct experiments in space and the laboratory, and to study muscle atrophy from a broader perspective with multiple useful genetic tools. Here, we showed *C. elegans* muscle cell area decreased in two independent experiments aboard the ISS. The physiological phenotype we observed in muscle is consistent with previous reports showing gene expression declines in key muscle genes in *C. elegans*, including *myo-3* and *unc-54* myosin heavy chain genes, and the *unc-15* paramyosin gene, from two independent space experiments [19,20,37]. Indeed, the downregulation of myosin heavy chain and paramyosin genes was consistent with observed decreases in protein levels in the same spaceflight experiments [20] as well as previous spaceflight experiments [15,38]. These data together with our observations suggest that space microgravity regulates *C. elegans* muscle cell size by altering muscle gene expression [39,40].

Our methodology to assess the size of the dorsal lateral muscle cells is based on phalloidin fluorescence staining of muscle actin and determining the approximate borders of each muscle cell. In addition, we measured the maximal longitudinal area of each rhomboid-shaped muscle cell, ignoring the height dimension of each cell. Our methodology gives an approximate measurement of muscle cell volume and atrophy observed during spaceflight. Experimental limitations with the space samples prevented us from conducting more accurate methods, such as EM experiments, to assess muscle cell volume. In addition, *C. elegans* body length differences in space, as reported previously [20,41], can indirectly contribute to smaller muscle. Instead, we normalized muscle area with body length by calculating the MBR in all our experiments.

Although our initial MME mission samples showed a decline in muscle cell area in space, this experiment was hampered by low-quality ground controls for the WT, and we produced a laboratory control that mimicked spaceflight conditions. However, we confirmed our results from MME in the NIS experiments, which included a 1 G control sample aboard the ISS in addition to a 0 G sample. In these experiments, both the control and experimental samples experienced the same spaceflight launch, handling by astronauts and timing and conditions aboard the ISS. However, some of the conditions between MME and NIS were different. This included the cultivation time aboard the ISS (5 days in total for MME, 12 days in total for NIS; see Figure 1), the conditions of the medium (the chemical FUdR was added at 7.5 days to prevent egg laying of the F1 generation; see Methods) and the fixation conditions (−80 °C for MME, PFA fixation and 4 °C for NIS). Overall, these differences did not change the developmental time or quality of muscle and phalloidin staining, and we have confidence in the differences in muscle area and body length in both MME and NIS experiments.

Nutritional deprivation results in muscle atrophy in humans, rodents and flies [5,6,27]. Starvation in *C. elegans* is a large source of stress for adult animals, resulting in multiple physiological and behavior changes [42]. Interestingly, starvation does not result in death but rather an increase in *C. elegans*’ lifespan [43,44], presumably through a starvation resistance pathway that alters metabolism [42]. However, the effects of starvation on muscle and body length have not been assessed. Here, we show that longitudinal muscle cell area and body length decreased substantially within 5 h of starvation at the L4 stage, and muscle cell size continued to decline after 48 h of starvation (Figure 6A). Octopamine regulates starvation resistance in adult animals as well as other physiological and behavioral aspects of starvation, and the nuclear receptor *daf-12* and the octopamine biosynthesis gene *tbh-1* promote starvation resistance [35,42,45,46]. We wondered whether *tbh-1* and *daf-12* could also mediate nutritional deprivation-induced muscle atrophy, but *tbh-1* and *daf-12* mutants showed a similar starvation-induced MBR to the WT, demonstrating that another starvation-resistance pathway may be regulating these changes (Figure 7A,B).

Disuse of muscle, as observed in bedridden patients, patients with spinal cord injury and spinal cord denervation and hind-limb suspension experiments in rodents, results in muscle atrophy [4,9,28,29]. We used a *C. elegans* mutant, *unc-13 (s69)*, to simulate muscle disuse experiments in the nematode. UNC-13 protein is necessary for synaptic vesicle release, and *unc-13 (s69)* mutants are almost completely paralyzed because no evoked synaptic activity occurs at the neuromuscular junction [32]. Mutations in genes more specific to the neuromuscular junction, such as acetylcholine receptor genes *unc-17* and *cha-1*, may be more appropriate; however, null mutants of these genes are so severe that they induce larval developmental arrest [47,48,49]. *unc-13* mutants had a smaller body wall muscle area and decreased MBR compared to WT animals (Figure 7A,B), according to results obtained using the standard method. 

However, since *unc-13 (s69)* mutants have a complete loss of synaptic activity throughout the body and have some neurosecretory defects [50], we cannot completely rule out the possibility that other neural factors may cause smaller muscle cell size.

Comparing muscle atrophy in space microgravity with nutritional deprivation and disuse, we found a decrease in the MBR of between 20% and 30% under space microgravity conditions, and a roughly 40% decline in both starvation and disuse conditions (Figure 6E). In addition, body wall muscle shape was almost always preserved in the control under each of these atrophy-inducing conditions (Figure 3 and Figure 6B). This is in contrast to the altered shape observed in the disruption of core proteins involved in muscle attachment complexes, which results in a complete collapse of the sarcomere structure into ball-like structures [34] that we never observed in our experiments. Although we occasionally observed spacing between the phalloidin-stained actin of adjacent muscle cells and slight alterations in shape in nutritionally deprived and disuse animals (Figure 6B), these qualitative phenotypes were rare and inconsistent. Overall, the muscle atrophy observed in space microgravity was not greatly different from that induced by nutritional deprivation or disuse.

In our candidate genetic screening, we found that mutation in the *clp-4* gene suppressed starvation-induced muscle atrophy from over 43% of the MBR to less than half at 21% of the MBR. Calpains are Ca^2+^-dependent proteases that exhibit limited proteolytic activity and are ubiquitously present [51,52]. They are known to be involved in apoptosis, muscle protein turnover and muscle maintenance in general [34,53,54]. Humans have three *clp-4* orthologs, CAPN1 (calpain 1), CAPN2 (calpain 2) and CAPN3 (calpain 3), which have been implicated in limb-girdle muscular dystrophy [55,56]. Calpains play a role in muscle protein degradation when attachment complexes are disrupted, and CLP-4 is expressed in muscle cells and required to maintain arrayed attachment complexes [34,56]. How then can CLP-4 promote starvation-induced muscle atrophy? We speculate that nutritional deprivation leads to cell and ER stress, resulting in release of ER calcium stores and activation of the Ca^2+^-dependent protease, but further analysis will be required to substantiate this. Furthermore, we would like to eventually confirm whether *clp-4* or calpain-mediated proteolysis also plays a role in promoting space-microgravity-induced muscle atrophy.

## 4. Materials and Methods

### 4.1. Strains and Culture

The *C. elegans* strains used in this study were cultivated and maintained on Nematode Growth Medium (NGM) plates seeded with *E. coli* OP50 as the food source. The NGM plates were prepared using the standard method [57]. The strains were obtained from the Caenorhabditis Genetics Center (CGC; Minneapolis, MN, USA), the National BioResource Project (NBRP; Tokyo, Japan) and through plasmid injection. The strains used included the Bristol N2 wild-type strain, RB2123 *clp-4 (ok2808)*, RB1728 *tra-3 (ok2207)*, RB2084 *clp-7 (ok2750)*, DM2 *dim-1 (ra102)*, AA86 *daf-12 (rh61rh411)*, MT9455 *tbh-1 (n3247)*, CB1091 *unc-13 (s69)*, CB524 *unc-70 (e524) I*, CZ13799 N2; *juIs76* obtained from CGC, *clp-1 (tm690)* obtained from NBRP and YUW105 *unc-70 (e524)*; *juIs76* from our laboratory.

### 4.2. Conditions for MME Space Experiment

To determine a suitable liquid culture for the MME mission that would preserve the worms’ integrity when frozen but not affect the worms’ development and population growth, different freezing solutions with varying concentrations and combinations were tested. Synchronized L1 larva-stage worms were prepared and transferred into polyethylene bags containing LabTie *E. coli* OP50 (Biovirid, Veenendaal, The Netherlands) dissolved in buffer (S-basal + cholesterol, 2× final LabTie concentration) with or without cryoprotectant solution and incubated for 6 days at 12 °C (to mimic the storage temperature prior to arrival at the ISS) and another 6 days at 20 °C (actual incubation temperature aboard the ISS). Culture bags were then retrieved, and worms’ developmental and growth states were checked under a dissecting microscope. Then, they were frozen at −80 °C. After several days, culture bags were thawed, and worms were again observed for GFP integrity and signals. We found that dimethyl sulfoxide (DMSO) and trehalose treatment optimally preserved the GFP quality from the samples compared to sorbitol treatment or no cryoprotectant treatment (Table S1). However, it does not provide ideal development and population growth for *C. elegans*. Thus, a lower concentration of trehalose solution was tested under our MME space sample conditions, and the worms’ survival, development and population growth were observed regularly under a dissecting microscope. However, 0.08 mM trehalose treatment still conferred a slightly negative effect on the growth of the worms (Table S2). Thus, we proceeded with S-basal with cholesterol without any cryoprotectant as the appropriate culture condition for the MME space experiment.

### 4.3. MME Space Experiments

The MME space mission was conducted in collaboration with the European Space Agency (ESA). We initially determined the proper conditions for spaceflight, and the samples were prepared using a method similar to the one described previously by Laranjeiro et al. (2021) and Sudevan et al. (2022) [58,59]. The eggs were harvested by bleaching gravid worms following the standard protocol [60] on Day −1. On Day 0, approximately 500 synchronized L1 larvae were transferred to polyethylene flight culture bags containing S-basal (for 1 L S-basal: NaCl 5.85 g, K_2_HPO_4_ 1 g, KH_2_PO_4_ 6 g), cholesterol (5 mg/mL in EtOH stock; 1 ml for 1 L) and 2× concentration LabTie freeze-dried OP50 (Biovirid, Veenendaal, The Netherlands), totaling a volume of 6.5 mL per bag. These samples were then sealed and placed into experiment cassettes (ECs, Kayser, Toscana, Italy; [18]) and kept in cold stowage at temperatures ranging from 8 to 13 °C until they arrived at the International Space Station (ISS). After five days of incubation in the ISS, the samples were frozen at −80 °C until analysis. The control samples were prepared in the same manner as the space samples and handled and stored at NASA Kennedy Space Center (Cape Canaveral, FL, USA) or in the laboratory for lab WT.

### 4.4. NIS Space Experiments

The NIS space mission was conducted in collaboration with the Japan Aerospace Exploration Agency (JAXA). Approximately 200 synchronized L1 larvae were prepared on Day −2, added to polyethylene flight culture bags with M9 buffer with cholesterol (KH_2_PO_4_ 3 g, Na_2_HPO_4_ 12H_2_O 15.1 g, NaCl 5 g, 1 M MgSO_4_ 1 mL and 1 mL of cholesterol (5 mg/mL in EtOH) for a total of 1 L) and stored at 12 °C until the launch. When the samples arrived at the International Space Station (ISS), they were transferred to 20 °C incubators. The astronaut responsible for handling the samples took half of each sample and fixed them with 0.7% paraformaldehyde (PFA) when the F1-generation worms reached Day 1 and Day 10 of adulthood. To prevent starvation and the mixing of generations, the astronaut added media M9 buffer with cholesterol and *E. coli* food with FUdR (Sigma-Aldrich F0503, St. Louis, MO, USA, 75 μM), which interferes with DNA synthesis and prevents reproduction, after extracting the portion of samples. In this paper, only Day-10 adult samples were used for analysis. Control samples were also sent to the ISS and placed in a centrifuge that simulated a 1 G gravity force.

### 4.5. Starvation and Disuse Experiments

A total of 150 μL of S-complete (for 15 mL of S-complete: 14.61 mL of S-basal, 0.15 mL of 1 M potassium citrate, 0.15 mL of trace metals solution, 0.045 mL of 1 M CaCl_2_, 0.045 mL of 1 M MgSO_4_ and 0.015 mL of cholesterol; [57]) with LabTIE freeze-dried OP50 (0.9 g/250 mL) was added to each well of a 96-well plate (30096, SPL). To prevent rapid evaporation and ensure normal gas exchange, the 96-well plate was covered with a gas-permeable film (A9224, Sigma-Aldrich) instead of its plastic cover. L1-synchronized larvae were grown on *E. coli* OP50-seeded NGM plates at 20 °C for 48 h. These worms were then washed with S-basal to remove as much OP50 as possible from their bodies and divided into two groups, one with food and the other without food, and transferred to a liquid culture plate. After an additional 30 h of incubation, the samples were used for analysis. Each well contained fewer than 20 worms.

### 4.6. Phalloidin Muscle Staining

A previously described method for muscle staining, as outlined in [61], was used in this study. All samples were fixed with 4%PFA prior to staining. Following PFA fixation, the samples were washed with 1× PBS, and acetone was added for 1 min to enhance the permeability of phalloidin within the worms. Subsequently, the samples were washed again and treated with phalloidin conjugated with a fluorescent molecule. After overnight incubation at 37 °C, the samples were mounted onto glass slides covered with a 2% dry agarose pad. Observation and image capture were conducted using an Olympus BX50 microscope (Tokyo, Japan). All samples were stained with Alexa Fluor™ 488 Phalloidin (A12379, Invitrogen™, Waltham, MA, USA), which emits a green fluorescent signal, except for the NIS samples, which were stained with Rhodamine Phalloidin (R415, Invitrogen™), which emits a red fluorescent signal.

An adult *C. elegans* possesses 95 body wall muscles distributed along its body. These muscles are organized into four longitudinal bundles, one in each quadrant. Each bundle consists of staggered pairs of muscles, and the muscle cells within a bundle are numbered from 1 to 23 or 24, as described previously [62]. For this study, we specifically focused on measuring seven dorsal lateral body wall muscles located in the mid-body region, which are numbered 10, 12, 14, 16, 18, 20 and 22 (Figure 1). To measure the longitudinal muscle cell area and body length, a previously published protocol was used [21]. Briefly, the ImageJ software V1.52 was utilized as a measurement tool. The muscle-to-body length ratio, MBR, was calculated by dividing individual worms’ average muscle cell area by body length. Statistical analysis of the obtained data was conducted using Student’s *t*-test for space missions and one-way ANOVA followed by Tukey’s post hoc analysis (IBM SPSS, USA) for starvation and disuse experiments to determine the significance of the results.

## Data Availability

Data will be made freely available upon request to the corresponding author J.I.L.

## Supplementary Materials

The following supporting information can be downloaded at: https://www.mdpi.com/article/10.3390/ijms241612640/s1.

## Abbreviations

*C. elegans*	*Caenorhabditis elegans*
ISS	International Space Station
ESA	European Space Agency
WT	Wild type
NASA	National Aeronautics and Space Administration
MME	Molecular muscle experiment
NIS	Neural integration system
MBR	Muscle-to-body length ratio
EM	Electron microscope
FUdR	Fluorodeoxyuridine
PFA	Paraformaldehyde
GFP	Green fluorescent protein
DMSO	Dimethyl sulfoxide
EC	Experiment cassette
JAXA	Japan Aerospace Exploration Agency
