# Supplementary material for: Comparative Analysis of Muscle Atrophy During Spaceflight, Nutritional Deficiency and Disuse in the Nematode Caenorhabditis elegans
Source: Int J Mol Sci. 2023 Aug 10;24(16):12640. doi: 10.3390/ijms241612640 (PMC10454569; doi:10.3390/ijms241612640)
Supplement: Supplementary file 1 [file ijms-24-12640-s001.zip › ijms-2542178-supplementary.pdf]

Supplemental Materials

Supplemental Methods

Worm Liquid Culture and Preservation Condition for MME

To determine a suitable liquid culture for the MME mission that will preserve the worm's integrity when frozen but will not affect the worm's development and population growth, different freezing solutions with varying concentrations and combinations was tested. Synchronized L1s were prepared and transferred in a JAXA PE bags containing LabTie *E. coli* OP50 dissolved in buffer (2x final concentration) with or without cryoprotectant solution and incubated for 6 days at 12°C (to mimic the storage temperature prior to arrival at ISS) and another 6 days at 20°C (actual incubation temperature aboard the ISS). Culture bags were then retrieved, and worm's conditions were checked under a dissecting microscope, then were frozen at -80°C. After several days, culture bags were thawed and worms were again observed for GFP integrity and signals. We found that dimethyl sulfoxide (DMSO) and trehalose optimally preserved the GFP quality from the samples (Table S1). However, it does not provide ideal development and population growth for *C. elegans*. Thus, a lower concentration of trehalose solution was tested under our MME space sample conditions, and worm's survival, development and population growth was observed regularly under a dissecting microscope. A final decision to use the S-basal with cholesterol without any cryoprotectant to was made for the actual MME space experiment (Table S2).

Supplemental Tables

Table S1. List of cryoprotectant solutions tested and the condition. ("+" good, "++" excellent, "-" poor).

Cryoprotectant	Development and Population Growth	GFP Quality
M9 Buffer	++	-
S-basal Cholesterol	++	-
2.5% Sorbitol Sol'n. (w/o NaCl)	+	-
5% Sorbitol Sol'n. (w/o NaCl)	+	-
16% Sorbitol Sol'n.	-	Not tested
1% Me_2SO	+	-
1.5% Me_2SO	+	Not tested
2.5% Me_2SO	-	Not tested
3% Me_2SO	-	++
5% Me_2SO	-	++
1.5% Me_2SO 1% Glycerol	-	Not tested
2.5% Me_2SO 1% Glycerol	-	Not tested
1.5% Me_2SO - 1% Trehalose	-	+
1.5% Me_2SO - 3% Trehalose	+	+
2.5% Me_2SO - 1% Trehalose	+	+
2.5% Me_2SO - 2% Trehalose	+	+

2.5% Me_2SO - 2.5% Trehalose	+	++
2.5% Me_2SO - 3% Trehalose	-	+
2.75% Me_2SO - 2% Trehalose	+	+
2.75% Me_2SO - 2.5% Trehalose	+	++

Table S2. Comparison of worms' development growth on S-basal with cholesterol and with trehalose.

Duration	Temperature	S-Basal With Cholesterol	S-Basal with Cholesterol + 0.08m Trehalose
Day 0	12°C	L1	L1
Day 4	12°C	L4	L4
Day 7 (Day 0 at 20°C)	20°C	Young adult	Young adult
Day 9 (Day 2 at 20°C)	20°C	Adult, many eggs, L1 and L2	Adult, many eggs, L1 and L2
Day 11 (Day 5 at 20°C)	20°C	Majority adult worms	Majority L3-L4
Day 12 (Day 6 at 20°C)	20°C	+ 40,000 worms total	+ 40,000 worms total
